# Impact of Probiotics Administration on the VEGF, Adiponectin, and Glycolipid Metabolism, in Prediabetic Patients: A Randomized, Double‐Blinded, Placebo‐Controlled Clinical Trial

**DOI:** 10.1002/fsn3.70146

**Published:** 2025-05-01

**Authors:** Mehrdad Sarabi, Ashkan Torshizian, Zahra Mazloum Khorasani, Abdollah Firoozi, Hassan Mehrad Majd, Nastaran Khoshhal, Nikoo Saeidi, Mina AkbariRad

**Affiliations:** ^1^ Student Research Committee, Faculty of Medicine Mashhad University of Medical Sciences Mashhad Iran; ^2^ Metabolic Syndrome Research Center Mashhad University of Medical Sciences Mashhad Iran; ^3^ Department of Endocrinology, Faculty of Medicine Mashhad University of Medical Sciences Mashhad Iran; ^4^ Mashhad University of Medical Sciences Mashhad Iran; ^5^ Molecular Medicine, Faculty of Medicine Mashhad University of Medical Sciences Mashhad Iran; ^6^ Student Research Committee Islamic Azad University Mashhad Iran; ^7^ Department of Internal Medicine, Faculty of Medicine Mashhad University of Medical Sciences Mashhad Iran

**Keywords:** adiponectin, prediabetes, probiotic, resistin, VEGF

## Abstract

It is debated that probiotics can improve glycolipid metabolism and slow the progression of prediabetes to diabetes mellitus. This study aimed to evaluate the effect of probiotics on lipid profile, glucose homeostasis, serum level of resistin, adiponectin, and vascular endothelial growth factor (VEGF) in prediabetic patients. This double‐blind, randomized, placebo‐controlled clinical trial was conducted on prediabetic patients in the Endocrinology clinic of Ghaem Hospital. Patients were randomly divided into two groups: the probiotics group was prescribed a daily 500‐mg capsule of probiotics (10^9^ colony‐forming units), while the other received a placebo capsule with the same appearance. After 3 months, the effect of probiotic administration on laboratory parameters indicative of glycolipid metabolism, resistin, adiponectin, VEGF, body mass index (BMI), and blood pressure was compared between groups. This study was registered in the Iranian Registry of Clinical Trials (IRCT 20190801044405 N2). Fifty‐two patients were included in the final analysis, with 26 patients in each group. The mean age of patients was 43.75 ± 8.45. At the beginning, both groups were similar in all demographic characteristics and measured serum levels of investigated biomarkers (*p* > 0.05 for all parameters). Both groups exhibited significant changes in BMI and fasting blood sugar (FBS). However, regarding FBS, the magnitude of change was significantly greater in patients treated with probiotics (*p* = 0.022). Our findings also revealed a significant increase in HDL (*p* = 0.001), adiponectin (*p* < 0.001), and VEGF (*p* = 0.024) serum levels and a significant decrease in HbA1c (*p* = 0.034), LDL (*p* = 0.002), TG (*p* < 0.001), and total cholesterol (*p* = 0.001) exclusively in the probiotics group. Probiotic supplementation efficiently improved glycolipid metabolism, adiponectin, and VEGF serum levels.

## Graphical abstract

Probiotics improve metabolic markers in prediabetes patients.

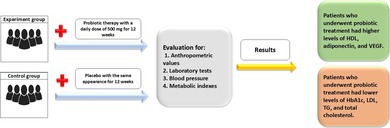

## 1 Introduction

Diabetes mellitus (DM) type 2 and prediabetes are major public health concerns, and their prevalence and incidence have substantially increased (Galicia‐Garcia et al. 2020). Prediabetes is characterized as a hyperglycemic state in which the glucose levels do not meet the criteria for DM (Tabák et al. 2012). Prediabetes is more common than type 2 DM (Zand et al. 2018). Between 2007 and 2010, around 34.1% of the world population was prediabetic, but it is predicted that by 2030, over 470 million individuals will be diagnosed with this condition (Tabák et al. 2012). The hallmarks of prediabetes and type 2 DM are gradual changes in insulin synthesis and secretion from the pancreas and alterations in insulin action in the liver, adipose tissue, and skeletal muscle (Daniele et al. 2014). Approximately 40%–50% of prediabetic patients progress to the diabetic stage in 10 years; given the notably increased risk of cardiovascular disease (CVD) in both diabetic and prediabetic patients, early detection and treatment of prediabetes are of great importance (DeFronzo and Abdul‐Ghani 2011; Mellbin et al. 2010).

A large number of biomarkers have been implicated to play a role in the progression of prediabetes to diabetes, amongst which are adiponectin and resistin (Jamaluddin et al. 2012; Yanai and Yoshida 2019; Zhou et al. 2021a). These markers are also known to alter the risk of CVDs in these affected populations. Relatively, vascular endothelial growth factor (VEGF) is known to play an important role in cardiac ischemic preconditioning and cardiac muscle health that can reduce the risk of CVD development and the complications following an acute myocardial infarction (Maulik 2004).

Resistin, also known as adipocyte‐specific hormone, plays a significant role in atherosclerosis, diabetes, insulin resistance, and obesity (Zhou et al. 2021a). Studies indicate its potential involvement in pathological CVD‐causing processes such as inflammation, angiogenesis, thrombosis, endothelial dysfunction, and smooth muscle cell dysfunction (Jamaluddin et al. 2012). Furthermore, vascular endothelial growth factor (VEGF) is a crucial component in the course of CVDs. The VEGF family has been proposed as a potential treatment for CVDs due to their role in the regulation of angiogenesis, inflammation, vascular permeability, lymphangiogenesis, apoptosis, and fibrogenesis (Zhou et al. 2021b). Adiponectin, a peptide secreted solely by mature adipose tissue, is another important biomarker affecting both glycolipid metabolism and cardiovascular health (Yanai and Yoshida 2019). Low serum concentrations of adiponectin are strongly associated with increased incidences of type 2 DM and heightened CVD risk (Thorand et al. 2010).

Beyond these biomarkers, recent research has highlighted the significant impact of gut microbiota composition on metabolic disorders and cardiovascular health (He and Shi 2017). Imbalances of gut microbiota (dysbiosis) are more common in diabetic patients compared to healthy individuals, and a balanced intestinal microbiota could help prevent diabetes (Sato et al. 2017). Consequently, probiotics have been proposed as a potential treatment to enhance metabolic health and prevent type 2 DM or prediabetes by altering intestinal microbiota and controlling insulin signaling (Hulston et al. 2015; Upadhyaya and Banerjee 2015).

Probiotics are live microorganisms that have been shown in various trials to delay the progression of DM and improve glucose homeostasis (Andersson et al. 2010; Ejtahed et al. 2012; Yadav et al. 2007; Yadav et al. 2008; Yun et al. 2009). The most prevalent bacteria with probiotic properties are *Lactobacillus* and *Bifidobacterium* strains, which are present in various dietary supplements (Sáez‐Lara et al. 2016).

The rapidly increasing incidence of diabetes mellitus and its burden on health systems and society prompted us to conduct this study. We aimed to investigate the potential effect of probiotics on glycolipid metabolism, cardiovascular health, and the concentrations of biomarkers that play a role in the progression of prediabetes to diabetes in prediabetic patients.

## 2 Materials and Methods

### 2.1 Study Design and Population

#### 2.1.1 Study Settings

This double‐blind, randomized, placebo‐controlled clinical trial was conducted on prediabetic patients from January 2020 to January 2022 in the Endocrinology clinic of Ghaem Hospital, affiliated with Mashhad University of Medical Sciences, Mashhad, Iran.

#### 2.1.2 Sample Size and Calculations

VEGF and adiponectin serum levels were the primary outcomes of this study. However, it is important to note that at the time of sample size calculation, there were limited or no available studies for Adiponectin, while some data were accessible for VEGF. Therefore, the sample size calculation for this trial was primarily based on a study by Szulińska et al. (Szulińska et al. 2018), utilizing the parameters of a significance level (alpha) of 0.05 and a power of 90%. Consequently, the sample size for each arm of the study was initially determined to be 19 patients for each outcome. To accommodate potential dropouts or unforeseen circumstances, a final sample size of 26 patients was enrolled in each arm of the study. The decision to consider adiponectin as a pilot outcome was based on the absence of relevant prior research, and this designation was made to guide future investigations and to provide context for the study's design and sample size calculations.

#### 2.1.3 Randomization

Given our study's relatively small sample size, patients were assigned to each group (placebo or probiotic) using a computer‐based block randomization approach (fixed block size of 4). To avoid allocation bias, each patient was given sealed envelopes determining the patient's study group. Once randomly assigned, a physician outside the main research team opened the envelopes and prescribed probiotics or placebos accordingly. The physicians assessing patients at baseline and upon follow‐up were blinded to their allocation.

#### 2.1.4 Treatment Groups

The probiotic group was prescribed a daily 500‐mg capsule of probiotics (produced by Zist Takhmir Pharmaceutical Co.) equal to 10^9^ colony‐forming units (CFU). This dosage is considered the minimal effective dose of probiotics that can change the composition of the gut microbiome (Office of Dietary Supplements 2024). At the same time, the other group received a placebo capsule with the same weight and appearance.

The prescribed probiotic capsules contained the following bacterial strains: *Lactobacillus rhamnosus, Lactobacillus helveticus, lactobacillus casei, Bifidobacterium lactis, lactobacillus acidophilus, Bifidobacterium breve, Lactobacillus bulgaricus, Bifidobacterium longum, Lactobacillus plantarum, Bifidobacterium bifidum, Lactobacillus gasseri*, and *streptococcus thermophilus*.

#### 2.1.5 Lifestyle Modifications

Regardless of the assigned group, all patients were advised to follow comparable lifestyle recommendations, including exercise and a high‐fiber, low‐calorie diet. A medical intern contacted all participants weekly to ensure they followed the instructions. Our initial study protocol indicated that patients be excluded if they deviated from their instructed lifestyle modification protocols.

### 2.2 Inclusion and Exclusion Criteria

We included newly diagnosed prediabetic patients aged between 30 and 65 years. Prediabetes was defined as any of the following conditions:

1. Fasting blood glucose between 100 and 125
2. Glycated hemoglobin (HbA1c) of 5.7% to 6.4%
3. An oral glucose tolerance test 2‐h serum glucose level of 140–199 mg/dL

Patients with a declined glomerular filtration rate (less than 60 mL/min), a previous history of CVD, gastrointestinal malabsorption, chronic diarrhea, and secondary obesity were excluded. We also excluded patients who had received antihyperglycemic agents, probiotics, antibiotics, aspirin, or group B vitamins in the last 3 months. A plant‐based diet, pregnancy, smoking, and a previous diagnosis of prediabetes or diabetes were additional exclusion criteria of our study. The exclusion criteria remained consistent during the patients' follow‐up, meaning that if any patient developed one of the above‐mentioned conditions or decided to discontinue treatment, he or she was excluded from our analysis.

## 3 Outcomes

### 3.1 Data Collection

Patients' demographic data, body mass index (BMI), systolic and diastolic blood pressure, and laboratory test results were collected using an institutional form at baseline and after 3 months of follow‐up. Laboratory examinations included testing serum levels for insulin, low‐density lipoprotein (LDL), high‐density lipoprotein (HDL), triglycerides (TG), resistin, fasting blood sugar (FBS), HbA1c, VEGF, and adiponectin.

### 3.2 Primary Outcomes

The primary outcomes of this study were VEGF and adiponectin levels.

### 3.3 Secondary Outcomes

Insulin resistance was evaluated in all patients at baseline and 3 months later using the Homeostatic Model Assessment for Insulin Resistance (HOMA‐IR) (Matthews et al. 1985) scoring system. At the same time, the risk of atherosclerotic cardiovascular disease (ASCVD) was assessed according to the 2019 ACC/AHA guideline (Arnett et al. 2019) at baseline and 3 months later.

### 3.4 Statistical Analysis

The distribution of preintervention and postintervention continuous variables was assessed using the Shapiro–Wilk test. Normally distributed data were presented as mean and standard deviation, and the comparison between preintervention and postintervention data was made using a paired *t*‐test in these cases. Continuous variables with abnormal distribution were presented as median and interquartile range, and a comparison between preintervention and postintervention data was made using the Wilcoxon signed‐rank test. The continuous variables were compared between the groups at the beginning of the study using either the Mann–Whitney *U*‐test or independent samples *t*‐test according to their distribution, while gender differences were assessed using a Chi‐squared test. A *p* < 0.05 was considered statistically significant. All analyses were conducted in IBM SPSS statistics V 26.

### 3.5 Ethical Consideration

Written informed consent was obtained from all patients, according to the World Medical Association Declaration of Helsinki, revised in 2000, Edinburgh. Moreover, the study protocol was fully approved by the Ethics Committee of Mashhad University of Medical Sciences (IR.MUMS.MEDICAL.REC.1399.650). This study was registered in the Iranian Registry of Clinical Trials with the IRCT number: IRCT 20190801044405 N2.

## 4 Results

**FIGURE 1:**
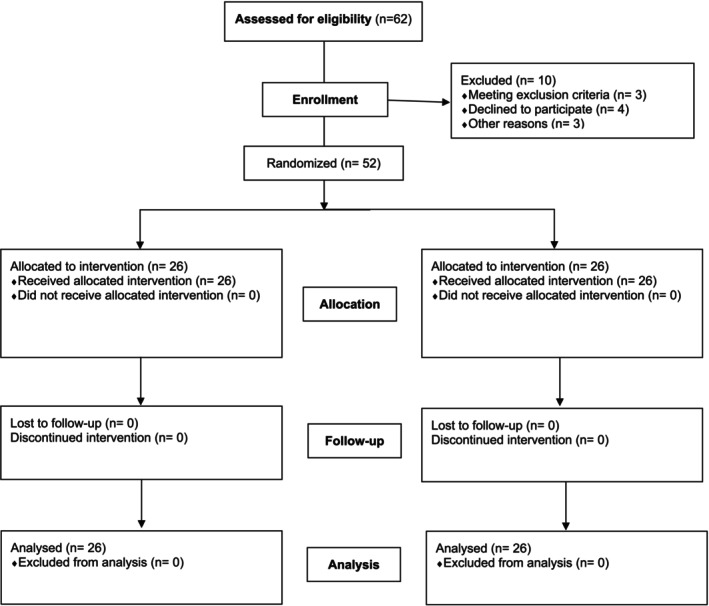
Study participants' flow chart.

A total of 62 patients were initially assessed for inclusion in our study, of which 10 were excluded due to different causes (Figure 1). The remaining (*N* = 52) were randomized into two groups (placebo and probiotic). Upon study completion, no dropouts were observed and no patients were excluded according to the exclusion criteria.

The mean age of patients was 43.75 ± 8.45, and the majority of our study population consisted of females (71.1%).

At the beginning of the trial, the two groups did not significantly differ regarding their demographics (age and BMI), blood pressure, and baseline laboratory examinations, including FBS, HBA1c, insulin, TG, LDL, HDL, resistin, VEGF, and adiponectin levels (Table 1). ASCVD risk and insulin resistance were also compared between the two groups and did not differ statistically (*p* > 0.05 for all parameters).

**TABLE 1:** Comparison of demographic and basic biomarker serum levels between the intervention and control groups at the beginning of the study.

Variables	Intervention group	Control group	*p*
Age (year)*	(50.0‐37.0) 42.0	(51.0‐35.0) 41.0	0.503
Gender			
Male (%)	7 (46.2)	8 (53.8)	0.760
Female (%)	19(51.6)	18 (48.4)	
BMI (kg/m2)*	(34.02‐28.72) 29.51	(33.17‐28.67) 29.51	0.981
Systolic blood pressure (mmHg)*	(130.0‐120.0) 122.5	(130.0‐120.0) 122.5	0.839
Diastolic blood pressure (mmHg)*	(85.0‐70.0) 80.0	(90.0‐80.0) 85.0	0.065
FBS (mg/dl)*	(114.0‐104.0) 107.5	(116.0‐103.0) 106.0	0.510
HbA1c (mg/dl)**	0.64 ± 5.66	0.98 ± 5.68	0.928
Insulin (μIU/mL)*	(15.9‐7.6) 11.2	(25.0‐4.4) 10.05	0.869
TG (mg/dl)**	42.96 ± 152.18	53.33 ± 141.86	0.484
LDL (mg/dl)**	26.10 ± 122.09	46.04 ± 110.23	0.301
HDL (μIU/mL)*	(48.0‐39.0) 45.5	(50.0‐40.0) 46.0	0.410
Total Cholesterol**	196.8 ± 29.39	185.41 ± 47.30	0.343
Resistin (μg/mL)*	0.09 (0.07‐0.11)	0.09 (0.08‐0.10)	0.597
VEGF (μg/mL)*	0.17 (0.15‐0.17)	0.16 (0.15‐0.18)	0.503
Adiponectin (μg/mL)**	0.15 ± 0.02	0.16 ± 0.02	0.194
ASCVD risk (%)*	39 (36‐39)	39 (27‐40.75)	0.917
HOMA‐IR*	3.13 (1.93‐4.31)	2.56 (1.21‐6.54)	0.833

Abbreviations: ASCVD, atherosclerotic cardiovascular disease; BMI, body mass index; FBS, fasting blood sugar; HDL, high‐density lipoprotein; HOMA‐IR, homeostatic model assessment for Insulin resistance; LDL, low‐density lipoprotein; TG, triglyceride; VEGF, vascular endothelial growth factor.

* Values are shown as median (interquartile range), and the Mann–Whitney *U*‐test was used for comparison.

** Values are shown as mean ± standard deviation, and independent *t*‐test was used for comparison.

Table 2 compares various characteristics of patients in the placebo group at the beginning and end of the study. There were no significant changes in the levels of resistin (*p* = 0.845) and adiponectin (*p* = 0.055) in the placebo group. However, the patients' BMI and FBS declined significantly after 3 months (*p* < 0.001, and *p* = 0.006, respectively). The same attributes were compared in patients in the probiotic group at the beginning and end of the trial (Table 3). Similar to the placebo group, BMI (*p* < 0.001) and FBS (*p* < 0.001) significantly decreased in the probiotic group. We also observed additional significant changes in other assessed parameters in this group. Serum levels of VEGF (*p* = 0.024), HDL (*p* = 0.001) and adiponectin (*p* < 0.001) significantly increased, while levels of LDL, TG, HbA1c, and ASCVD risk scores significantly declined.

**TABLE 2:** Comparison of different variables in the placebo group before and after the intervention.

Variables	Before intervention	After intervention	*p*
BMI (kg/m^2^)*	(33.17–28.67) 29.51	(32.28–26.79) 28.65	0.001 >
Systolic blood pressure (mmHg)*	(130.0–120.0) 122.5	(131.25–120.0) 125.0	0.870
Diastolic blood pressure (mmHg)*	(90.0–80.0) 85.0	(89.25–78.75) 82.5	0.372
FBS (mg/dl)*	(116.0–103.0) 106.0	(106.25–89.0) 100.5	0.006
HbA1c (mg/dl)*	(5.97–4.97) 5.70	(6.02–4.97) 5.65	0.354
Insulin (μIU/mL)*	(25.0–4.4) 10.05	(21.05–5.6) 11.35	0.961
TG (mg/dl)*	(173.0–104.0) 138.0	(169.75–87.0) 117.0	0.999 <
LDL (mg/dl)**	46.04 ± 110.23	31.87 ± 116.50	0.373
HDL (μIU/mL)**	10.08 ± 46.82	9.23 ± 48.50	0.364
Total cholesterol	186.1 (147.55–212.3)	201.6 (166.2–214.05)	0.299
Resistin (μg/mL)*	0.09 (0.07–0.11)	0.09 (0.09–0.11)	0.845
VEGF (μg/mL)**	0.16 (0.15–0.18)	0.16 (0.15–0.19)	0.225
Adiponectin (μg/mL)**	0.16 ± 0.02	0.15 ± 0.02	0.055
ASCVD risk (%)*	39 (27–40.75)	39 (27–40.75)	0.747
HOMA‐IR*	2.56 (1.21–6.54)	2.89 (1.51–4.68)	0.910

Abbreviations: ASCVD, atherosclerotic cardiovascular disease; BMI, body mass index; FBS, fasting blood sugar; HDL, high‐density lipoprotein; HOMA‐IR, homeostatic model assessment for Insulin resistance; LDL, low‐density lipoprotein; TG, triglyceride; VEGF, vascular endothelial growth factor.

* Wilcoxon test was used.

** A paired *t*‐test was used.

**TABLE 3:** Comparison of different variables in the intervention group before and after the intervention.

Variables	Before intervention	After intervention	*p*
BMI (kg/m^2^)*	(34.02–28.72) 29.51	(33.01–26.78) 29.09	0.001 >
Systolic blood pressure (mmHg)*	(130.0–120.0) 122.5	(130.0–120.0) 125.0	0.528
Diastolic blood pressure (mmHg)**	7.76 ± 79.32	7.47 ± 80.23	0.710
FBS (mg/dl)**	6.28 ± 109.59	5.80 ± 93.50	0.001 >
HbA1c (mg/dl)*	(6.10–5.32) 5.80	(5.72–4.77) 5.35	0.034
Insulin (μIU/mL)*	11.2 (7.x27‐16.5)	11.5 (7.42–17.0)	0.650
TG (mg/dl)**	42.96 ± 152.18	33.55 ± 109.05	0.001 >
LDL (mg/dl)**	26.10 ± 122.09	23.63 ± 95.27	0.001
HDL (μIU/mL)**	6.26 ± 44.27	5.79 ± 49.64	0.001
Total Cholesterol	200.2 (174.65–221.7)	158.0 (147.15–190.35)	0.001
Resistin (μg/mL)**	0.09 ± 0.01	0.16 ± 0.23	0.176
VEGF (μg/mL)*	0.17 (0.15–0.17)	0.20 (0.16–0.23)	0.035
Adiponectin (μg/mL)**	0.15 ± 0.02	0.17 ± 0.02	< 0.001
ASCVD risk (%)*	39 (36–39)	36 (27–39)	0.049
HOMA‐IR*	3.13 (1.93–4.31)	3.86 (1.82–5.98)	0.140

Abbreviations: ASCVD, atherosclerotic cardiovascular disease; BMI, body mass index; FBS, fasting blood sugar; HDL, high‐density lipoprotein; HOMA‐IR, homeostatic model assessment for insulin resistance; LDL, low‐density lipoprotein; TG, triglyceride; VEGF, vascular endothelial growth factor.

* Wilcoxon test was used.

** A paired *t*‐test was used.

At last, we compared 3‐month changes of the assessed parameters in the placebo and probiotic groups (Table 4), as well as their serum levels between the two groups at the end of the follow‐up period (Table 5). Both changes and absolute amounts of ASCVD risk score, FBS, TG, LDL, total cholesterol, VEGF, and adiponectin varied significantly between the probiotic and the placebo groups. Regarding other parameters, no significant difference was found between the two.

**TABLE 4:** Comparison of different variable changes between the intervention and control groups.

Variables	Intervention group	Control group	*p*
Δ BMI (kg/m^2^)**	−0.76 (−0.37 _ −1.32)	−0.77 (−0.40 _ −1.33)	0.925
Δ Systolic blood pressure (mmHg)**	5.0 (10.0 _ −5.0)	−5.0 (15.0 _ −10.0)	0.776
Δ Diastolic blood pressure (mmHg)*	0.90 ± 11.30	0.18 ± 14.51	0.854
Δ FBS (mg/dL)*	−16.09 ± 7.35	−8.31 ± 13.21	0.022
Δ HbA1c (mg/dL)*	−0.34 ± 0.73	−0.13 ± 0.99	0.421
Δ Insulin (μIU/mL)**	2.5 (14.9_0.0)	0.6 (4.2 _ −6.7)	0.073
Δ TG (mg/dL)**	−37.50 (−20.0 _ −59.0)	−7.50 (48.0 _ −49.0)	0.012
Δ LDL (mg/dL)*	−26.81 ± 30.89	6.27 ± 32.30	0.001
Δ HDL (μIU/mL)*	5.36 ± 6.27	1.68 ± 8.49	0.109
Δ Total cholesterol	−30.08 ± 34.84	9.70 ± 36.11	0.001
Δ Resistin (μg/mL)**	−2.00 (−4.25 _ −1.00)	−2.00 (−4.00 _ −1.00)	0.158
Δ VEGF (μg/mL)*	0.02 ± 0.00	0.01 ± 0.00	0.041
Δ Adiponectin (μg/mL)**	0.017 ± 0.019	0.009 ± 0.02	< 0.001
Δ ASCVD risk (%)*	−3 (−12 _ 0)	0 (−5.5 _ 10)	0.042
Δ HOMA‐IR*	0.37 (−0.35_3.42)	0.00 (−2.10 _ 0.91)	0.139

Abbreviations: ASCVD, atherosclerotic cardiovascular disease; BMI, body mass index; FBS, fasting blood sugar; HDL, high‐density lipoprotein; LDL, low‐density lipoprotein; TG, triglyceride; Homa‐IR: homeostatic model assessment for insulin resistance; VEGF, vascular endothelial growth factor.

* Mann–Whitney *U*‐test was used for comparison.

** An independent *t*‐test was used for comparison.

**TABLE 5:** Comparison of different variables between intervention and placebo groups postintervention.

Variables	Intervention group	Control group	*p*
BMI (kg/m^2^)*	(33.01–26.78) 29.09	(32.28–26.79) 28.65	0.851
Systolic blood pressure (mmHg)*	(130.0–120.0) 125.0	(131.25–120.0) 125.0	0.962
Diastolic blood pressure (mmHg)*	7.47 ± 80.23	(89.25–78.75) 82.5	0.262
FBS (mg/dl)*	5.80 ± 93.50	(106.25–89.0) 100.5	0.042
HbA1c (mg/dl)**	(5.72–4.77) 5.35	(6.02–4.97) 5.65	0.365
Insulin (μIU/mL)*	11.5 (7.42–17.0)	(21.05–5.6) 11.35	0.133
TG (mg/dl)**	33.55 ± 109.05	(169.75–87.0) 117.0	0.079
LDL (mg/dl)**	23.63 ± 95.27	31.87 ± 116.50	0.016
HDL (μIU/mL)*	5.79 ± 49.64	9.23 ± 48.50	0.473
Total Cholesterol**	158.0 (147.15–190.35)	201.6 (166.2–214.05)	0.013
Resistin (μg/mL)*	0.16 ± 0.23	0.09 (0.09–0.11)	0.280
VEGF (μg/mL)*	0.20 (0.16–0.23)	0.16 (0.15–0.19)	0.037
Adiponectin (μg/mL)**	0.17 ± 0.02	0.15 ± 0.02	0.016
ASCVD risk (%)*	36 (27–39)	39 (27–40.75)	0.02
HOMA‐IR*	3.86 (1.82–5.98)	2.89 (1.51–4.68)	0.833

Abbreviations: ASCVD, atherosclerotic cardiovascular disease; BMI, body mass index; FBS, fasting blood sugar; HDL, high‐density lipoprotein; HOMA‐IR, homeostatic model assessment for insulin resistance; LDL, low‐density lipoprotein; TG, triglyceride; VEGF, vascular endothelial growth factor.

* Mann–Whitney U‐test was used for comparison.

** An independent t‐test was used for comparison.

## 5 Discussion

### 5.1 Effects of Probiotics on Glycemic State and Glucose Metabolism

We investigated the possible impact of probiotic administration on patients' glycemic state and glucose metabolism after 3 months. For this purpose, conventional lab examinations, including FBS, HbA1c, and fasting insulin levels, were obtained at the beginning and end of the trial. Our analysis demonstrated a meaningful decrease in FBS levels of both probiotic and placebo groups. When the magnitude of this decrease was compared between the two groups, the analysis revealed a more significant decrease in FBS among patients receiving probiotics. HbA1c also dropped significantly in the probiotic group. The individuals receiving placebo also experienced a slight drop in this measure, but no statistically significant changes were observed. No significant changes in fasting insulin levels were observed among either group. FBS improvements in both groups are most likely due to patients' lifestyle modifications and dietary changes, as all patients were occasionally contacted and advised to maintain a low‐calorie diet and daily exercise. Given that only the probiotic group experienced significant changes in HbA1c, and that the probiotic group exhibited a significantly larger decrease in FBS, it can be perceived that the ingestion of probiotics, in addition to dietary and lifestyle changes, can be more effective than dietary and lifestyle changes alone in the regulation of glucose homeostasis and preventing the progression of prediabetes to diabetes. Insulin resistance was also evaluated in all patients prior to and after the trial using HOMA‐IR. This indicator did not exhibit significant changes in either group. Given the observed beneficial effects of probiotics on other factors indicative of glucose metabolism, the absence of changes in patients' insulin resistance might be due to the short follow‐up period since this index was recalculated 3 months after the initial evaluation. As changes in gut microbiome composition and production of short‐chain fatty acids (SCFAs) by this altered microbiota are among the major pathways through which probiotics affect glucose metabolism (Delzenne and Cani 2011), it is worth mentioning that changes in gut microbiota may occur over a more extended period, and a longer follow‐up period may be necessary to detect the effects of probiotics administration on insulin resistance (Oh et al. 2021). Oh, et al. and Naito et al. also evaluated this index in prediabetic subjects 8 weeks after probiotic supplementation and found insignificant changes in HOMA‐IR (Naito et al. 2018; Oh et al. 2021).

In their recent systematic review and meta‐analysis of the available trials on the potential benefits of probiotics in prediabetes, Li et al. concluded that probiotics could effectively improve blood glucose homeostasis by lowering levels of HbA1c (Li et al. 2022), which is consistent with our findings. Nevertheless, they claimed that the data supporting the impact of probiotics on the decline in FBS and HOMA‐IR were inconclusive. Further research is, thus, required to better understand this issue, given the observed effect of probiotics on FBS reduction and the unexpected absence of changes in HOMA‐IR in our study population.

### 5.2 Effects of Probiotics on Lipid Profile

Regarding the lipid profile, significant changes were observed among all associated parameters in the probiotic group, while no changes occurred in the placebo group. Total cholesterol, TG, and LDL significantly decreased in patients receiving probiotics, while HDL levels increased dramatically in this group. The beneficial impact of probiotics on lipid metabolism can be explained by a number of mechanisms, including the hydrolysis of bile salts into nonabsorbable free bile acids (Toshimitsu et al. 2019), inhibition of cholesterol resynthesis through the production of SCFAs (Naito et al. 2018), and reduction of cholesterol absorption by incorporation of cholesterol into their cell membranes, and conversion of cholesterol into fecal sterols that can be excreted into the feces (Lye et al. 2010). Following a meta‐analysis of four trials on the potential effects of probiotics in prediabetes patients, Li et al. concluded that probiotics therapy could effectively reduce TG and total cholesterol, which is in line with our study. However, previous studies have failed to show significant changes in HDL following the administration of probiotics (Naito et al. 2018; Yan et al. 2023), while our findings revealed a substantial increase in this biomarker. As all of these studies, including ours, were comprised of small sample sizes, further research into this matter is necessary to obtain a comprehensive understanding of probiotics' impact on lipid profile.

### 5.3 Effects of Probiotics on VEGF, Resistin, and Adiponectin

Serum concentrations of VEGF and resistin were also calculated among all participants at the beginning and end of the trial. Our analysis demonstrated that VEGF levels rose significantly in the probiotics group. Resistin levels, however, did not exhibit significant changes in either group. Various studies have reported normal VEGF serum concentrations in healthy individuals between small amounts below the detection limit to a maximum level of 0.99 μg/L (Raimondo et al. 2001; Riedel et al. 2000). Given the fact that the serum concentration of this biomarker did not exceed 0.21 μg/L in either group of this study, it can be assumed that all participants had normal levels of VEGF.

Increased VEGF‐A levels within the normal range favor the proliferation of vascular endothelial cells (VECs), improve vascular permeability, and restore the integrity of the endothelium and vascular function. Therefore, it compensates for ischemia and hypoxia and protects the injured myocardium (Zhou et al. 2021b). We found no other study assessing this biomarker in prediabetic patients. However, Szulińska et al. assessed VEGF levels 3 months after the administration of probiotics in postmenopausal obese women. They observed that lower doses of probiotics, as defined by their study method (2.5 × 10^9^ CFU, which is closer to our administered dose of 10^9^ CFU), had no impact on VEGF levels. In comparison, higher doses were associated with a decrease in VEGF. However, their subjects differed in various characteristics, and comparing their study with ours may not be rational. Given the hypothesized positive effect of VEGF on cardiovascular health and the observed increase of this biomarker in prediabetics receiving a placebo, further research on probiotics' effect on VEGF serum levels and the impact of VEGF serum levels on cardiovascular health in prediabetics is crucial.

Our findings revealed a significant increase in serum adiponectin levels in patients treated with probiotics. Adiponectin has been identified as a peptide with noteworthy positive effects on glycolipid metabolism, insulin sensitivity, and cardiovascular health (Yanai and Yoshida 2019). These effects have been explained through various pathways affecting multiple organs. Suppression of inflammation and alleviation of oxidative stress are known to be major pathways through which adiponectin contributes to improving insulin resistance and cardiovascular health. This peptide reduces the secretion of pro‐inflammatory and inflammatory cytokines such as CRP and TNF‐α (Ouchi et al. 2000; Ouchi and Walsh 2007). Modulation of macrophages' phenotype and function is another mechanism through which this hormone reduces inflammation (Libby et al. 2002). Other known mechanisms involved in regulating glycolipid metabolism and preventing atherosclerotic disease by adiponectin consist of its protective effects on functional pancreatic B cells, inhibition of hepatic gluconeogenesis and glycogenolysis, and increased glucose uptake and utilization by skeletal muscle and adipose tissue (Yanai and Yoshida 2019). Previous studies have also shown an inverse correlation between serum adiponectin levels with TG and LDL, while it is positively correlated with serum HDL (Cnop et al. 2003). Unlike other adipokines, adiponectin seems to be negatively correlated with the portion of adipose tissue, meaning that obesity results in reduced secretion of this hormone (Cnop et al. 2003). BMI decrease was present in both groups of our study, with no significant difference between the two groups. However, the elevation of adiponectin was only observed in the probiotic group. This implies that another factor contributing to this increase might be in play. We hypothesized that this increase is due to the potential role of probiotics in reducing inflammation and oxidative stress, as secretion of adiponectin is markedly reduced in the presence of proinflammatory cytokines (Yanai and Yoshida 2019). To the best of our knowledge, Toshimitsu et al. are the only other group that has assessed the effects of probiotics on adiponectin in prediabetes patients. Similar to our findings, they also reported a significant increase in adiponectin levels following the administration of probiotics in prediabetic patients (Toshimitsu et al. 2019).

## 6 Conclusion

Probiotics significantly improved serum levels of various biomarkers, including FBS, HbA1c, total cholesterol, TG, LDL, HDL, adiponectin, and VEGF. The benefits of probiotics on glycolipid metabolism and its associated biomarkers have been discussed earlier in this study. As glycolipid metabolism and cardiovascular health are tightly associated, it is expected that the regulation of glycolipid metabolism by probiotics directly impacts and alleviates cardiovascular health. Our study followed patients only for 3 months. We suggest that future studies consider a more extended follow‐up on patients and assess the effects of probiotics on the incidence of type 2 diabetes mellitus and cardiovascular diseases.

### 6.1 Limitations and Strengths

The limitations of this study include a short 3‐month follow‐up period and a relatively small sample size, which was affected by the challenges of patient recruitment during the COVID‐19 pandemic. Additionally, the homogeneity of the study population and the lack of microbiome analysis limit a deeper understanding of the mechanisms through which probiotics affect glycolipid metabolism. Despite these limitations, the study's strengths lie in its robust randomized, double‐blind, placebo‐controlled design, minimizing bias and enhancing the validity of the results. The study also ensured regular follow‐up to monitor adherence to lifestyle modifications, such as diet and exercise, enhancing the reliability of the observed probiotic effects. The focus on clinically relevant biomarkers like adiponectin, VEGF, and lipid profiles provided valuable insights into the cardiometabolic effects of probiotics.

## Author Contributions

**Mehrdad Sarabi:** data curation (equal), investigation (supporting), project administration (lead), writing – original draft (supporting), writing – review and editing (equal). **Ashkan Torshizian:** formal analysis (supporting), investigation (supporting), software (supporting), writing – original draft (lead), writing – review and editing (equal). **Zahra Mazloum Khorasani:** resources (equal), supervision (equal). **Abdollah Firoozi:** investigation (supporting), resources (equal). **Hassan Mehrad Majd:** conceptualization (supporting), formal analysis (equal), software (lead). **Nastaran Khoshhal:** investigation (equal). **Nikoo Saeidi:** investigation (supporting), writing – original draft (supporting). **Mina AkbariRad:** conceptualization (lead), methodology (lead), project administration (supporting), supervision (lead).

## Disclosure

Approval date of Registry and the Registration No. of the study/trial: Our study was registered in the Iranian Registry of Clinical Trials with the number IRCT Number: IRCT 20190801044405N2 on 7/05/2021.

Animal studies: The authors have nothing to report.

## Ethics Statement

Approval of Research Protocol: Our current work has been approved by the Ethical Committee at Mashhad University of Medical Sciences under the code: IR.MUMS.MEDICAL.REC.1399.650.

## Consent

All patients signed informed consent.

## Conflicts of Interest

The authors declare no conflicts of interest.

## Data Availability

The data that support the findings of this study are available from the corresponding author upon reasonable request.
